# Reactive Astrocytes in Glioma: Emerging Opportunities and Challenges

**DOI:** 10.3390/ijms26072907

**Published:** 2025-03-23

**Authors:** Jiasheng Wu, Ran Li, Junwen Wang, Hongtao Zhu, Yixuan Ma, Chao You, Kai Shu

**Affiliations:** Department of Neurosurgery, Tongji Hospital, Tongji Medical College, Huazhong University of Science and Technology, 1095, Jie Fang Avenue, Qiao Kou District, Wuhan 430030, China; d202382331@hust.edu.cn (J.W.); jwwang@tjh.tjmu.edu.cn (J.W.); zhuhongtao@tjh.tjmu.edu.cn (H.Z.); mayx@hust.edu.cn (Y.M.)

**Keywords:** gliomas, reactive astrocytes, tumor microenvironments, immunity, glial molecular signaling

## Abstract

Gliomas are the most prevalent malignant tumors in the adult central nervous system (CNS). Glioblastoma (GBM) accounts for approximately 60–70% of primary gliomas. It is a great challenge to human health because of its high degree of malignancy, rapid progression, short survival time, and susceptibility to recurrence. Owing to the specificity of the CNS, the glioma microenvironment often contains numerous glial cells. Astrocytes are most widely distributed in the human brain and form reactive astrocyte proliferation regions around glioma tissue. In addition, astrocytes are activated under pathological conditions and regulate tumor and microenvironmental cells through cell-to-cell contact or the secretion of active substances. Therefore, astrocytes have attracted attention as important components of the glioma microenvironment. Here, we focus on the mechanisms of reactive astrocyte activation under glioma conditions, their contribution to the mechanisms of glioma genesis and progression, and their potential value as targets for clinical intervention in gliomas.

## 1. Introduction

The latest epidemiological reports indicate that the overall incidence of malignant brain tumors in adults is approximately 7 per 100,000 population, with an overall 5-year survival rate of 36% [1]. The overall percentage of gliomas is about 80-85%, with glioblastoma (GBM) accounting for 49% and diffuse lower-grade glioma (LGG) accounting for 30% among malignant brain tumors [2]. Currently, the most used clinical management paradigms for gliomas are surgery combined with radiotherapy and temozolomide chemotherapy. In the case of GBM, the 2-year and 5-year survival rates for combination therapy are 27.2% and 9.8%, respectively, significantly improved compared with 10.9% and 1.9% for monotherapy [1]. Even so, gliomas remain difficult to cure and are one of the great threats to human life and health.

As one of the fundamental hallmarks of cancer, the formation of the tumor microenvironment significantly contributes to the development and progression of gliomas [3,4]. In the early years, studies related to the glioma microenvironment were mainly focused on immune cells in the traditional sense (e.g., T and B cells, monocytes, dendritic cells, etc.) and microglia. As for astrocytes, they are widely present in the glioma microenvironment, and pathologists have found areas of astrocyte proliferation around glioma tissues. Since gliomas also contain tumors of astrocyte origin (also known as astrocytomas), the early focus of scientists tended to be on how to differentiate between tumorigenic and nontumorigenic astrocytes or to explore the mechanisms by which astrocytes malignantly transform into glioma cells [5,6,7]. However, this has also led to a relative underappreciation of the biological significance of astrocytes as members of the microenvironment in glioma genesis, progression, and evolution.

Reviewing the research data on reactive astrocytes, we see that their development has proceeded through several major stages. The origins of this scientific problem lie in a phenomenon that is often accompanied by lesions of the central nervous system (CNS) called “reactive astrogliosis”. As neuroscience progressed, scientists noticed that reactive astrocyte “scarring” was also present in a variety of neuroimmune and degenerative diseases such as multiple sclerosis, Alzheimer’s disease, and Parkinson’s disease. As a result, the neurotrophic and supportive capacity of astrocytes has been implicated in the neuronal degeneration and deficits of these diseases, and abnormal reactive astrocytes in pathological states are thought to be neurotoxic. For example, referring to the macrophage/microglia M1/M2 typing strategy, reactive astrocytes can also be roughly classified into two phenotypes named A1 and A2 [8,9], with the A1 phenotype considered neurotoxic and the A2 phenotype having neuroprotective functions such as the promotion of axonal injury repair and neuronal regeneration [8,10]. Notably, the model of pathology induction in A1 relies on cytokines such as *TNF-α*, *IL-1α*, and *C1q* secreted by microglia after lipopolysaccharide stimulation [8]. Also, multiple complement cascade regulators are upregulated in reactive astrocytes in A1. They can exert a wide range of regulatory effects on immune and non-immune cells via *fibroblast growth factor (FGF)*, *C-X-C Motif Chemokine Ligand 1 (CXCL1)*, *C-X-C Motif Chemokine Ligand 2 (CXCL2)*, *Interleukin 6 (IL6)*, etc. [8,10,11,12]. Conversely, A2 can inhibit the immune response by releasing TGF-β, protecting the CNS from damage due to excessive inflammatory responses [12]. Thus, astrocytes are essential regulators of inflammation in the CNS. Notably, immune escape stands as one of the defining hallmarks of gliomas. Some characteristic molecules of certain reactive astrocyte subsets have been widely investigated within the glioma context. For example, TGF-β plays a pivotal role in sustaining glioma proliferation, invasion, and immune evasion. It can achieve this by preserving the viability of glioma stem cells (GSCs), recruiting Tumor-Associated Macrophages (TAMs), and inducing the exhaustion of *CD8+* T cells and NK cells [13,14,15,16]. This has inspired oncologists to consider whether reactive astrocytes in the glioma microenvironment are merely “candidates” for carcinogenesis or whether they can act as organizers and participants in the formation and evolution of the glioma microenvironment.

Encouragingly, there is now a fundamental understanding that glioma-reactive astrocytes are widespread in the microenvironment and can exert pro-tumorigenic effects. Astrocytes activated by stimuli from glioma cells, microglia, and hypoxic conditions can enhance the proliferative, invasive, and metastatic capabilities of glioma cells through the secretion of effector molecules or via direct contact facilitated by gap junctions [17,18,19,20,21,22,23,24,25]. In organotypic slice models and in vivo mouse and rat models, the depletion of astrocytes has been significantly observed to cause a reduction in the in situ growth rates of gliomas [26,27,28]. Moreover, reactive astrocytes are linked to the generation of resistance to radiation and chemotherapy in gliomas [29,30]. They also correlate with the recurrence of gliomas after surgical resection [26,31]. Consequently, intervention strategies targeting reactive astrocytes hold promise as a potential adjuvant approach for the treatment of gliomas.

**Figure 1:**
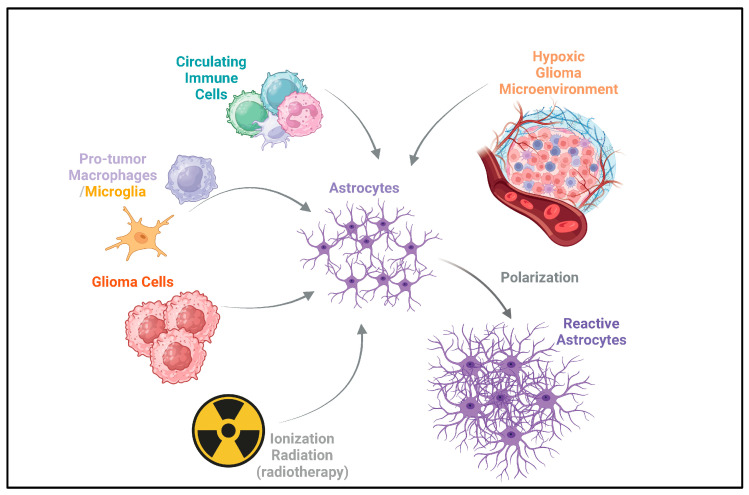
Acquisition of reactivity by astrocytes in glioma. Astrocytes can acquire reactivity through multiple ways, including circulating immune cells, pro-tumor macrophages/microglia, and glioma cells. Ionizing radiation from radiotherapy also acts on astrocytes [17,18,19,20,21,22,23,24,25,26,27,28]. This can potentially induce reactivity in astrocytes, which may contribute to the recurrence of gliomas after radiotherapy [30,32]. Additionally, the hypoxic glioma microenvironment drives the polarization of astrocytes, leading to the formation of reactive astrocytes [24]. The arrows depict the direction of influence and interaction among these components in the glioma microenvironment.

Here, we focus on the mechanisms of interaction between reactive astrocytes and microglia, circulating immune cells, and tumor cells in the context of glioma after having been validated in cellular or zoological models. At the same time, we focus on the mechanisms and heterogeneity of astrocytes acquiring reactivity in the glioma microenvironment and their potential value during clinical interventions in gliomas. Figure 1 briefly summarizes the acquisition of reactivity by astrocytes in the glioma microenvironment.

## 2. Astrocytes and Glioma Oncogenesis

There are three major viewpoints regarding glioma oncogenesis: the hypothesis of glioma stem cell origin, the hypothesis of neural stem cell origin, and the hypothesis of glial cell origin. The proposal, development, and refinement of these hypotheses largely rely on the development of single-cell RNA sequencing (scRNA-seq) technology and the advanced algorithms derived from it [33,34]. This enables us to further search for, subdivide, and define the sub-populations of glioma cells, as well as, to some extent, to infer their origins and directions of differentiation. Glioblastoma cells (WHO 4, IDH-wt) can be classified into four classic states based on scRNA-seq profiles and malignancy: oligodendrocyte progenitor cell-like (OPC-like), astrocyte-like (AC-like), neuronal progenitor cell-like (NPC-like), and mesenchymal-like (MES-like) subtypes [34]. In these subtypes, AC-like glioma cells express some characteristic astrocyte markers, such as *S100B*, *GFAP*, *SLC1A3*, *GLAST*, and *MLC1*. As is well known, tumor phenotypic transformation results from stage-specific transcriptional changes in progenitor cells. Currently, there is no clear definition of astrocyte precursors. However, under specific conditions, such as traumatic brain injury, astrocytes can redifferentiate and acquire the ability to enter the cell cycle for proliferation [35]. In addition, Li et al. mentioned in a cross-lineage analysis study that mouse cortical radial glial cells can give rise to *ASCL1⁺EGFR⁺* apical multipotent intermediate progenitor cells, which then differentiate into basal multipotent intermediate progenitor cells expressing *achaete-scute complex-like 1 (ASCL1)*, *epidermal growth factor receptor (EGFR)*, *oligodendrocyte transcription factor 2 (OLIG2)*, and *proliferating cell nuclear antigen Ki-67 (MKI67)*. These progenitor cells can later transform into astrocyte lineage-restricted progenitor cells [36]. This evidence indicates that astrocyte precursors may persist in the CNS and can differentiate to replenish astrocytes. Astrocytes may also redifferentiate under specific conditions, enhancing their proliferative capacity. These processes can be regarded as the event nodes of astrocytes. The oncogene and tumor suppressor gene mutations occurring therein may lead to glioma tumorigenesis. For example, in in vitro models, the in situ injection of astrocytes with oncogene and suppressor gene mutations can lead to the development of glioma. At the same time, the glioma cells formed in this way all express common astrocyte markers, such as *glial fibrillary acidic protein (GFAP)* [37,38,39]. Inducing the combined deletion of *phosphatase* and *tensin homolog (PTEN)*, *tumor protein p53 (TP53)*, and *retinoblastoma 1 (RB1)* in mouse astrocytes can lead to the malignant progression of glioma, manifested as a transition from WHO grade III to WHO grade IV [40]. These findings support the astrocyte origin part of the hypothesis of glial cell origin. However, it is too one-sided or incomplete to explain the origin of glioma with a single hypothesis. First, the hypothesis of glial cell origin cannot account for the origin of some glioma cell subsets, such as NPC-like cells. These cells characteristically express neural progenitor cell markers, such as *SOX4*, *SOX11*, and *DCX* [34]. Neural stem cells/progenitor cells are the starting point for the development of glial cells and nerve cells. Whether glioma cells originating from astrocytes can redifferentiate and form a phenotype like that of neural progenitor cells remains debatable. Furthermore, since neural progenitor cells can differentiate into the astrocyte lineage, does it mean that tumor cells with an astrocyte-like phenotype can originate from the downward differentiation of NPC-like cells? Secondly, GSCs, like NPC-like cells, also widely exist in glioma tissues of various pathological types and grades. They characteristically express stem cell factors such as *CD133, sex-determining region Y-box 2 (SOX2), Nestin,* and *OCT-4* [41]. These cells possess the abilities of self-renewal, differentiation, and resistance to DNA damage, and are important drivers of glioma progression [42,43]. Unfortunately, the glial cell origin hypothesis also fails to adequately explain the generation and formation of GSCs.

In summary, we tend to believe that the oncogenesis of gliomas may occur in the form of multiple cell sources, and glioma heterogeneity may not only result from the mutations or differentiation of a single cell line. The formation of glioma is a process of systemic breakdown in the CNS. The malignant transformation of astrocytes may be a crucial part of this process, or it could be the result of the accumulation of risk factors after the formation of basic glioma tissues. Elucidating these questions is significant for a clearer and deeper understanding of the glioma oncogenesis process.

Besides serving as a potential source of malignant cells, astrocytes have a robust ability to synthesize and secrete substances. This implies that non-malignant astrocytes can work together with glioma cells, microglia, macrophages, circulating immune cells, etc., to establish the glioma microenvironment and aid in the malignant progression of glioma [24,44,45,46]. These non-malignant astrocytes generally acquire reactivity upon receiving stimuli such as hypoxia and inflammatory factors in the microenvironment. Subsequently, we refer to them as “glioma-reactive astrocytes”.

## 3. Identification of Glioma-Reactive Astrocytes

Currently, the definition of glioma-reactive astrocytes is mainly based on findings in inflammatory diseases of the CNS. Common biomarkers include *GFAP*, *s100 calcium-binding protein B (S100B)*, *s100 calcium-binding protein A10 (S100A10)*, *complement component 1 q subcomponent (C1q)*, *neuroepithelial stem cell protein (NES)*, *vimentin (VIM)*, *and complement component 3 (C3)*. As early as 1978, astrocytes were shown to be more common in glioma tissues with relatively normal cell morphology typically distinct from malignant cells [47]. However, unlike the astrocytes in normal brain tissue, their volume is usually increased together with more branches. Although the concept of reactive astrocytes was lacking during that period, the authors described them as “well-preserved astrocytes”. Secondly, in an experimental model study, *GFAP* expression was also upregulated in astrocytes co-cultured with U87 [48]. *GFAP* is a crucial constituent of the astrocyte cytoskeleton. In the context of astrocytes undergoing reactive transformation, a conspicuous increase in both cell volume and the number of cell branches occurs. This reactive process is invariably accompanied by the upregulation of *GFAP* expression [49]. Thus, secretions from glioma cells can contribute to the acquisition of reactivity by astrocytes. Meanwhile, *transforming growth factor-β (TGF-β)*, *acid secretory protein (SPARC)*, and *matrix metallopeptidase 2 (MMP-2)* protein levels were also significantly elevated in U87 co-cultured astrocytes [48]. This further substantiates the existence of glioma-reactive astrocytes and indicates that they may be involved in the regulation of glioma immunity and the formation of an immunosuppressive microenvironment. *S100B* is an EF-hand-type Ca (2+)-binding protein highly expressed in astrocytes, which has various functions such as regulating Ca (2+)-homeostasis in the CNS and modulating cell proliferation and differentiation. Under pathological conditions, the S100B protein can regulate inflammatory responses through the MAPK and NF-κB pathways and mediate neurological damage by promoting oxidative stress [50,51]. In the context of gliomas, *S100B* can stimulate the phosphatidylinositol 3 -kinase/ protein kinase B (PI3K/Akt) and PI3K/RhoA pathways by interacting with *Rous sarcoma virus-related tyrosine-protein kinase src (Src kinase)*, which is involved in the activation of astrocytes [52]. *S100B* can also maintain immunosuppressive microenvironmental characteristics of gliomas by chemotaxis of TAMs through the upregulation of *CCL2* [53]. Notably, changes in the expression of markers such as *S100A10*, *C1q*, *NES*, *VIM*, and *C3* and their mechanisms of regulating reactivity have only been investigated in reactive astrocytes under non-tumor disease conditions [8,10,12]. In current experimental studies, the identification of glioma-reactive astrocytes also refers to the differential expression patterns of these markers. However, due to the specificity of gliomas, reactive astrocytes may be different in the context of gliomas than in general CNS inflammatory diseases. The activation mechanisms and the consequent effects of the expression of these markers in glioma-reactive astrocytes, along with the question of whether there exist more specific markers for glioma-reactive astrocytes, are all issues that merit further in-depth investigation.

The advancement and popularization of sequencing technologies (including bulk-/sc-/sn-RNA-seq, etc.) have provided new insights to address this issue. Al-Dalahmah et al. characterized the microenvironment of GBM using snRNA-seq and clustered GFAP-positive reactive astrocytes into three subtypes: protoplasmic astrocytes (AST1, highly expressing *SLC1A2/3/4*, *CPE*, *CPG5*), reactive astrocytes expressing oligodendrocyte and neuronal genes (*AST2*, high expression of *PLP1*, *RPL13A*, *RPL31*, *FTL*, *BCYRN1*), and reactive astrocytes expressing inflammatory genes (AST3, high expression of *S100B*, *CP*, *C3*, *CD44*, *CHI3L1/2*, *CLU*) [54]. By comparing with CNV-pos tumor cells, synaptic nucleoprotein genes (*SNCA*, *SNCB*, and *SNCG*), *WIF1*, *CHI3L2*, *ALDOC*, *ALDOA*, *AQP4*, carbonic anhydrases *CA2* and *CA11*, and *CXCL14* were characteristically highly expressed in reactive astrocytes. Henrik Heiland et al. performed a linear downscaling of astrocyte subpopulations in the GBM microenvironment after scRNA-seq sequencing based on the “Partitioning Around Medoids” algorithm. Six reactive astrocyte subtypes at different stages of differentiation were identified by gene set enrichment analysis [28]. Compared to simply using generic markers such as *GFAP*, *S100B*, and *VIM* for the identification of reactive astrocytes, this approach is currently relatively more concerned with the pathophysiological functions of the cells. This helps us to better understand and discover the functional localization of different reactive astrocyte subpopulations in the glioma microenvironment and the mechanisms behind their pro-tumorigenic effects. However, due to the limited sample size, the generalizability of these typing strategies is not yet known, and the academic community has not reached a consensus on this issue. Mechanisms of reactive astrocyte heterogeneity generation in the glioma microenvironment remain highly explorable in the future.

## 4. Interaction Between Reactive Astrocytes and Glioma Cells

Reactive astrocytes regulate the malignant progression of gliomas through several complex mechanisms. Most directly, reactive astrocytes can interact with tumor cells, thereby promoting their proliferation, invasion, and migration. This capacity depends on direct cell-to-cell contact mediated by membrane proteins on the surface of astrocytes or molecules delivered by non-contact means such as paracrine and extracellular vesicles. Secondly, normal informational interactions between astrocytes and other CNS components may be disrupted by gliomas, thereby facilitating their development and progression. Based on the available evidence, we initiated the review of this issue.

### 4.1. Abnormalities in Direct Cell-to-Cell Contact

Under physiological conditions, astrocytes have important CNS homeostatic maintenance functions, such as providing energy and nutrient support to neurons, balancing intra- and extracellular ionic and water homeostasis, scavenging neurotransmitters from synapses, participating in the formation and modulation of the blood–brain barrier, and regulating neuronal and synaptic plasticity based on gliotransmitter release [55,56]. These functions cannot be separated from the widespread physical connections between astrocytes or neurons/synapses, with gap junctions (GJs) dominated by the channel junction proteins *CX43* and *CX30* [57,58,59]. Pathologic changes in the CNS are often accompanied by abnormalities in the function of the material information exchange channels formed by the GJs. For example, during the formation of epileptic foci, disruption of GJ coupling in the astrocyte network leads to a reduction in the buffering capacity of potassium ions and the accumulation of intracellular sodium ions. Excess extracellular potassium ions contribute to the acquisition of abnormal excitability of neurons, and the accumulation of intracellular sodium ions in astrocytes further impairs the clearance of glutamate within the synapse, exacerbating the abnormal excitability of neurons [60,61]. Interestingly, altered membrane potential in cancer cells appear to be associated with glioma progression. For example, Venkatesh HS et al. discovered that non-synapse-dependent potassium currents can depolarize glioma membranes and promote their proliferation [62]. Notably, epilepsy is a common clinical manifestation of gliomas. In terms of pathogenesis, both glioma and epilepsy have been reported to be accompanied by the disorder of calcium ion regulation in astrocytes [63]. Thus, gliomagenesis might be potentially related to the disruption of astrocyte functional networks and extracellular ionic homeostasis. However, the relationship between homeostatic dysregulation associated with disruption of astrocyte functional networks and the development and progression of gliomas is still not fully elucidated. Current studies tend to be more inclined to focus on the abnormalities of glioma tissue but unconsciously ignore the subsequent effects caused by the disruption of normal CNS homeostasis. This issue deserves further exploration in the future.

**Figure 2:**
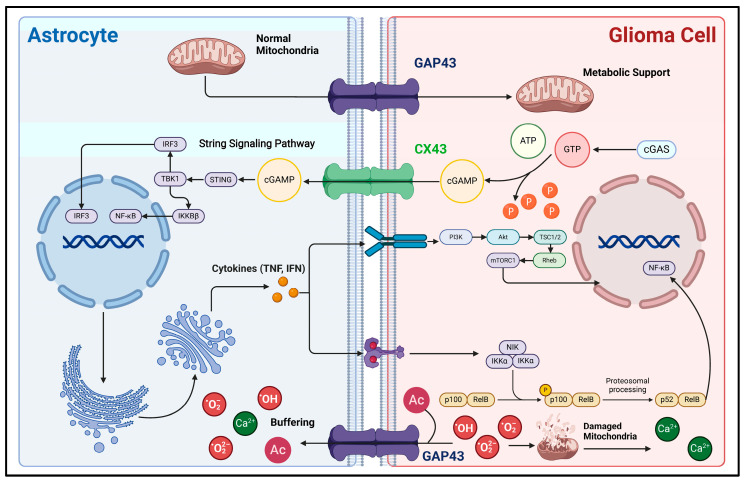
Mechanisms of direct material and information interaction between glioma cells and astrocytes. This figure illustrates the material interactions and associated signaling pathways between astrocytes and glioma cells via direct cell-to-cell contact. *CX43* and *GAP43* are important gap junction proteins between glioma cells and astrocytes, acting as bridges for the exchange of substances and information. During the rapid proliferation of glioma cells, due to their high demand for energy, their metabolic mode shifts to aerobic glycolysis as the main process. Astrocytes can transport normal mitochondria into glioma cells through *GAP43*, supporting their energy metabolism [18]. Also, aerobic glycolysis can generate a large amount of reactive oxygen species (ROS). First, ROS can cause mitochondrial damage, which releases a large amount of calcium ions, leading to calcium overload in glioma cells and subsequent apoptosis [70]. Second, the excessive accumulation of ROS itself is lethal. Glioma cells can transport these lethal factors into the astrocyte buffer pool network through *GAP43*, thereby developing resistance to cell death [69]. In addition, direct cell-to-cell contact is involved in the process of astrocytes acquiring reactivity. The signaling molecule cGAMP is produced within glioma cells and then transported into astrocytes via *CX43.* This activates the downstream STING and NF-κB signaling pathways, enabling astrocytes to acquire reactivity [20]. In this figure, the arrow represents the direction of the transmission of substances and signaling molecules.

Secondly, direct biphasic interactions of information and material between glioma cells and astrocytes can also be realized through GJs (Figure 2). Since 1999, glioma cells and astrocytes have been shown to produce physical connections via *CX43* under co-culture conditions. Also, the morphology of the corresponding astrocytes is significantly altered, as evidenced by an increase in cell volume and the number of membrane protrusions [64]. Sin et al. found that the knockdown of *CX43* in astrocytes resulted in a decrease in the migration of cancer cells from the core to the periphery of mouse glioma models, demonstrating that CX43-mediated reactive astrocyte-tumor cell signaling might be important in regulating the invasion and migration of gliomas [19]. Further studies have indicated that the second messenger cyclic guanosine monophosphate-adenosine monophosphate (cGAMP) can translocate from carcinoma cells into astrocytes via CX43-mediated cell–cell junctions, activate the STING pathway to increase their reactivity, and produce inflammatory factors such as *interferon-α (IFN-α)* and *tumor necrosis factor (TNF)* [20]. In turn, inflammatory factors can act as paracrine signals to activate STAT and NF-κB signaling pathways in carcinoma cells, thus supporting the malignant progression of gliomas [20,65]. In addition, *GAP43* is an actin-associated protein primarily responsible for regulating the formation of neural axon growth cones [66,67]. In astrocytes, *GAP43* appears to be associated with the formation and extension of cytomembrane protrusions. Osswald M et al. found that *GAP43* expression is upregulated in astrocytes in the context of gliomas, extends cytomembrane protrusions with *GFAP* as a supportive backbone, connects with glioma cells via *GAP43,* and constitutes a functional and resistant malignant cellular network. This network allows for the buffering of lethal damage within individual cells, such as the high levels of calcium release accompanying endoplasmic reticulum/mitochondrial damage [68]. It improves the viability of glioma cells. Furthermore, Watson et al. discovered that *GAP43* expression is upregulated in *GFAP+* astrocytes, potentially enhancing GBM tumorigenicity by facilitating mitochondrial transfer from reactive astrocytes to tumor cells through direct contact [18]. Thus, the GJ-dependent glioma–astrocyte network could increase the tolerance of gliomas during high growth rates, significantly improving their survivability and malignancy. Interestingly, *GAP43* can inhibit the activity of protein kinase and AKT signaling pathways in C6 glioma cells, demonstrating a tumor growth inhibitory effect. Typically, *GAP43* expression is absent in glioma cells [69]. Thus, *GAP43* can help distinguish glioma cells from reactive astrocytes.

### 4.2. Molecular Transfer via Non-Direct Contact

**Figure 3:**
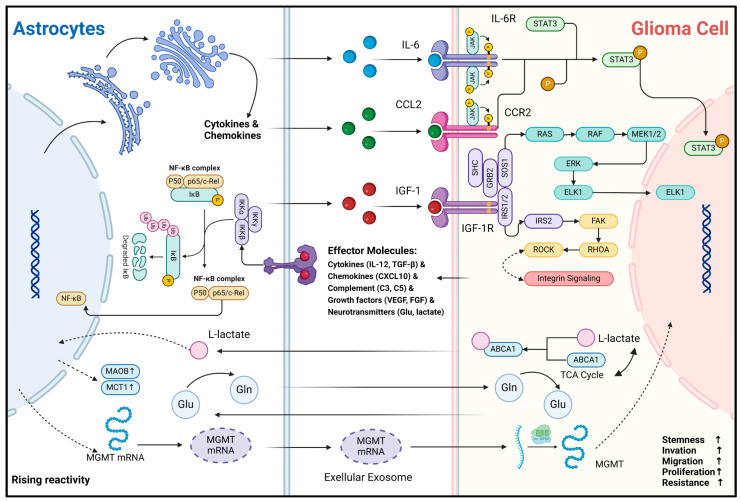
Mechanisms of non-direct material and information interaction between glioma cells and astrocytes. This figure illustrates the interaction and signaling mechanisms between astrocytes and glioma cells via non-direct contact. As the reactivity of astrocytes increases, the expression of molecules such as *MAOB* and *MCT1* rises, accompanied by metabolic changes in substances like L-lactate, glutamic acid (Glu), and glutamine (Gln). Gln can be synthesized by astrocytes and transported into glioma cells to support their metabolism. The metabolite of Gln, Glu, can then be transported back into astrocytes for the resynthesis of Gln, thus completing the Glu-Gln metabolic cycle [46]. In astrocytes, cytokines and chemokines are produced, and the NF-κB complex plays a crucial regulatory role [88]. Cytokines and chemokines secreted by astrocytes, such as *IL-6*, *CCL2*, and *IGF-1*, can act on the corresponding receptors (*IL-6R*, *CCR2*, *IGF-1R*, etc.) on glioma cells, activating downstream signaling pathways like JAK-STAT3 and RAS-RAF-MEK1/2-ERK and involving integrin signaling [22,23,45]. Glioma cells regulate their biological behaviors, including stemness, invasion, migration, proliferation, and resistance, through these signaling pathways. In addition, extracellular exosomes are involved in the information transfer between the two; for example, *MGMT* mRNA can be transported from reactive astrocytes to glioma cells via extracellular vesicles, contributing to the development of resistance to TMZ chemotherapy in glioma cells [80]. In this figure, the arrow represents the direction of the transmission of substances and signaling molecules.

Another important biological function of astrocytes is their ability to secrete a diverse range of effector substances upon sensing abnormal CNS events. In CNS inflammatory diseases, reactive astrocytes have been extensively studied. Their effector molecules are broadly categorized as follows: cytokines (e.g., *IL-1β*, *IL-6*, *TNF*, *IFN-γ*, *TGF-β*) [10,12,71], chemokines (e.g., *CCL2*, *CCL3*, *CCL5*, *CCL20*, *CXCL1*, *CXCL10*) [72], complement cascade components (e.g., *C3*, *C5*, *CFB*) [8], growth factors (e.g., *VEGFA*, *VEGFB*, *FGF*, *BMP1*, *NGF*, *BDNF*, *PDGFD*) [73], and neurotransmitters (e.g., Glutamate, ATP) [74,75]. The available evidence suggests that effector molecule secretion profiles of reactive astrocytes overlap between gliomas and non-tumorigenic CNS inflammatory diseases [45,76]. These molecules can bind to receptors on the surface or inside glioma cells, which in turn modulate the transformation of their malignant phenotype (Figure 3). For example, Oushy et al. found that extracellular vesicle components in glioblastoma cell culture medium supernatants stimulated astrocyte reactivity, which in turn upregulated the release of molecules such as *IL-12*, *IL-1A/B*, *CXCL10*, and *C5* [45]. These effector molecules can be directly recognized by glioma cells and play crucial roles in regulating their acquisition and maintenance of malignancy. *IL-6* can bind to *IL-6R* on the surface of glioma cells and activate the downstream JAK2/STAT3 signaling pathway, enabling them to acquire the stemness and invasiveness phenotype [22]. *IGF-1R* is widely present and highly expressed in glioma cells [77]. Exogenous *IGF-1* can enhance the invasive and migratory capacity of glioma cells by binding to *IGF-1R* and has been associated with resistance of gliomas to radiation, chemotherapy, and immunotherapy [25,77,78,79]. In addition, *CCL20* can be released by astrocytes to assist gliomas in counteracting hypoxia by binding to CCR6 to activate the NF-κB pathway and to upregulate HIF-1 expression [24]. Astrocyte-derived *CCL2* can bind *CCR2* on glioma cells to maintain stemness characteristics by activating JAK2/STAT3-Notch signaling pathway [23]. The effector molecules represented by cytokines and chemokines are relatively stable and can be delivered to glioma cells by paracrine secretion or extracellular vesicle trafficking. Notably, RNA transport between cells is a recent hot research topic. These less stable molecules and intracellular components can also be delivered into glioma cells and functionalized via extracellular vesicles or exosomes. A recent study indicates that mRNA for *O6-alkylguanine DNA alkyl transferase (MGMT)* is significantly elevated in exosomes from reactive astrocytes, enabling glioma cells to resist temozolomide (TMZ)-induced apoptosis by sequestering these exosomes [80]. However, only the phenomenon that glioma-associated astrocytes can produce and secrete these specific effector molecules is mentioned in these studies. Considering the holistic concept of the glioma microenvironment, other microenvironmental components such as microglia may also be part of the extra source of these effector molecules like *IL-6* and *CCR2* [81,82]. Therefore, the primary source of these effector molecules must be critically considered and investigated in the future. Fortunately, the rise of single-cell and spatial transcriptome sequencing technologies has provided effective ways to explore this question.

Secondly, metabolic and nutritional support is one of the important physiological functions of astrocytes. Astrocytes can generate metabolites such as L-lactate and L-serine via glycolysis and shuttle them to neurons to maintain energy requirements [83]. Also, astrocytes recycle toxic metabolites and neurotransmitters such as fatty acids and glutamate produced by neurons to protect normal neuronal function during periods of enhanced activity [55,84]. In the context of gliomas, astrocytes still fulfill their role as “metabolic transit stations”. L-glutamine (Gln) is crucial for maintaining carbon and nitrogen balance in neural tissues, primarily supplied by astrocytes in the CNS [85]. Under Gln-deficient conditions, the growth rate of gliomas is significantly inhibited [86]. Tardito et al. found that in GBM cells, almost half of the glutamate produced via the glutamine–glutamate cycle is secreted, but it does not enter the tricarboxylic acid (TCA) cycle. Instead of producing Gln for consumption, astrocytes synthesize Gln by uptake of glutamate and subsequently become the major provider of Gln for GBM growth metabolism [46]. Similarly, Perelroizen et al. found that reactive astrocytes can metabolically support GBM by delivering cholesterol extracellularly via *ABCA1* [27]. Conversely, acetic acid, an intermediate product of GBM metabolism, upregulates the expression of *MAO-B* and *MCT1* upon astrocyte uptake, in turn promoting the reactivity and proliferation of neighboring astrocytes [21]. In addition, glioma cells retain part of the metabolic characteristics of astrocytes, such as functioning in an aerobic glycolytic manner and producing the intermediate metabolite lactate. Under hypoxia and glucose deficiency, lactate can modulate astrocytes and reduce their inflammatory response [87]. Although the study was conducted in ischemic stroke models, the results align with our understanding of the glioma microenvironment, characterized by oxygen and nutrient deficiencies and immunosuppression. This gives us a hint that, in addition to the complementarity of metabolites, there is a synergy between reactive astrocytes and glioma cells in the process of microenvironment metabolic remodeling. The driving forces behind these metabolic remodeling and interaction processes in the oncogenesis and progression of gliomas, as well as the specific mechanisms by which subsequent effects are generated, deserve further in-depth exploration.

In summary, reactive astrocytes and glioma cells engage in various material and information exchange pathways, facilitating a mutual support that plays a crucial role in the mechanism of the malignant progression of gliomas.

## 5. Reactive Astrocytes and Remodeling of the Glioma Microenvironment

**Figure 4:**
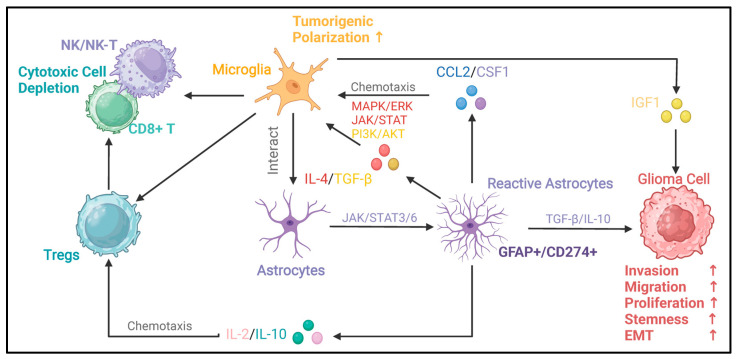
Oncogenic immunoregulatory functions of reactive astrocytes in the glioma microenvironment. This figure illustrates the oncogenic immunoregulatory functions of glioma-reactive astrocytes. Reactive astrocytes can secrete the chemokine *CCL2* and *CSF1* [27]. By activating the MAPK/ERK, JAK/STAT, and PI3K/AKT signaling pathways, they drive the polarization of microglia toward pro-tumor phenotypes [44]. Microglia with a pro-tumor phenotype can further promote the malignant progression of glioma cells through factors such as *IGF1*. Conversely, microglia can secrete factors such as *TGF-β* and *IL-10* [13,14,44]. By activating signaling pathways like JAK/STAT3/6, these factors enable astrocytes to acquire reactivity. Reactive astrocytes can secrete *TGF-β* and *IL-10*, which promote the invasion, migration, proliferation, stemness, and epithelial–mesenchymal transition (EMT) of glioma cells [90,91]. In addition, cytokines such as *IL-2* and *IL-10* secreted by reactive astrocytes can chemotaxis Tregs [24,28,44,45]. Tregs and microglia can interact with cytotoxic cells (NK/NK-T cells, CD8+ T cells), leading to the depletion of cytotoxic cells and immune-suppressive microenvironment [92,93]. In this figure, the arrows between different types of cells indicate the interaction relationships among the cells. The upstream of the arrow represents the regulator, and the downstream represents the responder. The arrow between astrocytes and reactive astrocytes represents the process and direction of polarization. The signaling molecules and signaling pathways in the middle describe the key signaling molecules and signaling pathways involved in these regulatory processes.

Astrocytes have multiple functions in the normal state, such as regulating water homeostasis, participating in the construction of the blood–brain barrier, constructing the extracellular matrix, and regulating angiogenesis and hemodynamics [89]. In the context of glioma, the ability of astrocytes to maintain homeostasis is disrupted, which may be related to the formation of the peritumoral edema zone, the development of tumor blood vessels, the remodeling of the extracellular matrix associated with invasion, and the breakdown of the blood–brain barrier. Secondly, astrocytes can secrete a variety of cytokines, which may be involved in the recruitment of cells that make up the microenvironment (Figure 4). Therefore, the potential mechanisms of the interaction between astrocytes and the glioma microenvironment deserve further attention.

### 5.1. Reactive Astrocytes and Glioma Angiogenesis

**Figure 5:**
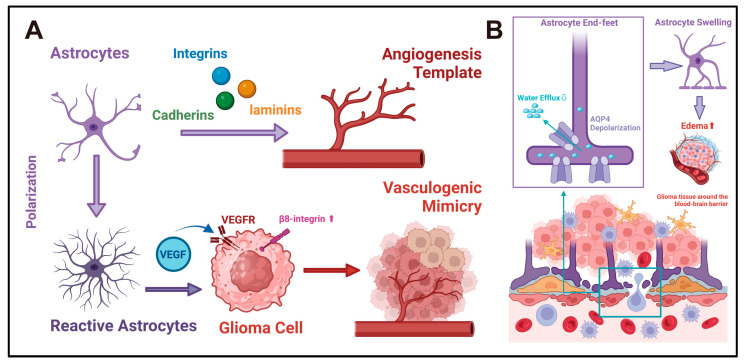
(**A**) Reactive astrocytes and angiogenesis in gliomas: Astrocytes may influence glioma angiogenesis via two potential mechanisms. First, astrocytes express diverse adhesion molecules, such as cadherins, integrins, and laminins, which offer attachment sites for endothelial cells, thereby serving as the template and framework for CNS angiogenesis [90,91]. Second, reactive astrocytes secrete angiogenic factors like *VEGF*. By facilitating the VM process, they may supply additional blood sources and invasion pathways for gliomas [101,102,103,104,107]. (**B**) Astrocytes and water homeostasis in gliomas: *AQP4* is the primary aquaporin in astrocytes and is localized to the astrocytic end-feet. It plays a crucial role in regulating cerebrospinal fluid circulation at the blood–brain barrier interface, thereby maintaining the water homeostasis of CNS [109,110]. In the pathological context, the depolarization of *AQP4* occurs at the end-feet of astrocytes [113]. This impedes the efflux of cerebrospinal fluid across the blood–brain barrier. As a result, the volume of astrocytes increases, and cerebrospinal fluid accumulates in the interstitial space. This might be one of the mechanisms underlying the development of glioma-associated edema. In this chart, the large arrows represent the direction of the functional evolution of cells or the interactions between cells. The signaling molecules in the middle of the arrows represent the key regulatory factors in this process. The small arrows represent the sites of action of the signaling molecules or the direction of the flow of substances.

During normal CNS development, astrocytes play an important role in the formation and development of blood vessels. Astrocytes can provide a growth framework for vascular endothelial cells and serve as a template for new blood vessels [94,95]. The disruption of the astrocyte template can lead to the formation of abnormal blood vessels. For example, selective interference with astrocytes in the retina can cause abnormal blood vessel networks [96]. To support their function as the angiogenesis scaffold, multiple cell adhesion molecules are present on the surface of astrocytes, such as cadherins, integrins, and laminins [97]. These adhesion molecules may provide potential sites for tumor angiogenesis. Notably, glioma cells retain characteristics like those of astrocytes and can express similar adhesion molecules on the surface. However, unlike astrocytes, tumor cells can rely on surface adhesion molecules to establish blood supply pathways by the unique mechanism of vasculogenic mimicry (VM). For example, *VE-Cadherin* is regulated by the transcription factor *FOXK1* and is highly expressed on the surface of glioma cells. It plays a crucial role in VM formation [98]. *β8 integrin (ITGB8)* can promote the formation of VM through the Smad2/3-RhoA signaling pathway in GBM [99]. Since VM provides channels for cancer invasion and metastasis, these surface adhesion molecules may be crucial pathways for the progression and acquisition of blood supply support of gliomas [100]. Secondly, astrocytes can secrete pro-angiogenic factors, including VEGF, FGF, and *angiopoietin* [101,102,103,104]. Under pathological conditions, the expression patterns of these factors will change. For example, under hypoxic conditions, *HIFs* can stimulate astrocytes to upregulate *VEGF* expression [105]. In ischemic stroke, *HIF-1α-*related signals can prompt reactive astrocytes to polarize towards the A2 phenotype. This can increase the expression of *VEGF* for promoting the formation of new blood vessels and protect the brain tissue from ischemia and hypoxia damage [106]. In the context of glioma, similar changes have also been reported. Vasiliki et al. found that reactive astrocytes in GBM tissues are localized near the peri-necrotic regions of *HIF-2α-*expressing cells and exhibit a robust hypoxic response. This is driven by *HIF-2α*, manifested as an elevation in the expression of hypoxia-related cytokines, including *TGF-β1*, *IL-3*, angiopoietin, *VEGFA*, and *IL-1α* [107]. In addition, some pro-angiogenic factors are also involved in the process of VM formation. For example, *VEGF* is also an important regulatory molecule of VM, and its expression is regulated by HIFs in the hypoxic microenvironment [108]. In summary, various evidence suggests that reactive astrocytes may directly participate in the regulation of glioma angiogenesis (Figure 5A). However, there are no direct experimental studies that have achieved a reduction in blood supply and inhibition of progression by intervening with glioma-reactive astrocytes. Angiogenic factors like *VEGF* are likewise expressed in glioma cells, microglia, etc. Whether the construction and remodeling of the glioma blood supply is dominated by astrocytes remains debatable. These issues deserve further exploration in the future.

### 5.2. Reactive Astrocytes and Disturbance of Water Homeostasis in Glioma

Astrocytes in the brain parenchyma are the main cell types expressing aquaporins. *Aquaporin 4 (AQP4)* is one of the important aquaporins on astrocytes, which is mainly enriched in the end-feet, located at the blood–spinal cord barrier and the blood–brain barrier, regulating water exchange on both sides [109,110]. It plays an important role in maintaining the stability of the blood–brain barrier, cell volume, and extracellular space volume of astrocytes [110]. Under pathological conditions, the expression and distribution of *AQP4* in reactive astrocytes will be altered. The glymphatic system promotes the reflux of cerebrospinal fluid and waste clearance in the brain during sleep through perivascular channels supported by glial cells [111,112]. In patients with Parkinson’s disease (PD), the localization of *AQP4* in the end-feet of astrocytes is reduced (*AQP4* depolarization), manifested as metabolic disorders in cerebrospinal fluid reflux and neurotransmitter clearance related to the impaired glymphatic system [113]. This exacerbates the loss of dopaminergic neurons. Similarly, *AQP4* depolarization also occurs in patients with Alzheimer’s disease (AD) and normal-pressure hydrocephalus. It is characterized by the accumulation of cerebral metabolic waste and amyloid-β plaques, as well as an increase in perivascular reactive astrocytes, which further accelerates the progression of cognitive impairment [114]. In a stroke model with ischemia and hypoxia, *AQP4* was overexpressed in reactive astrocytes around the infarct area. Targeted inhibition of *AQP4* could significantly alleviate post-infarct cerebral edema [115]. Cognitive impairment, as well as increased intracranial pressure and headaches caused by peritumoral tissue edema, are also common clinical symptoms in glioma. Therefore, an abnormal expression and localization of *AQP4* may exist in glioma and form part of the mechanism underlying the generation of clinical symptoms (Figure 5B). Existing evidence only mentions the increased expression of *AQP4* in glioma-reactive astrocytes but does not further clarify its specific mechanisms in regulating the flow of edema fluid in gliomas [116]. These issues deserve more profound and extensive research in the future.

### 5.3. Reactive Astrocyte–Microglia Interactions

Unlike other tissues, the CNS has unique immunity exemption mechanisms due to the presence of the blood–brain barrier. This results in the macrophage/microglia cells residing in the CNS being the predominant immune cell component in the general state. Thus, microglia may be among the first responders to abnormal events in the CNS [117,118]. Secondly, astrocytes are also prevalent in the CNS, so their immunomodulatory function has attracted attention. However, during the construction of reactive astrocyte models in the context of inflammation, it was found that although astrocytes express *TLR4*, the lack of intracellular response mechanisms (e.g., MYD88) prevented LPS stimulation alone from directly rendering astrocytes reactive [119,120]. However, cytokine-rich supernatants of microglia receiving LPS stimulation can induce A1 astrocytes in murine model [8]. This landmark discovery has directed attention to microglia–astrocyte interaction mechanisms. Recent studies indicate that astrocytes are crucial support cells for the growth and survival of microglia. For example, astrocyte-derived *IL-34*, *TGF-β*, and cholesterol molecules support branching microglia survival in vitro and maintain microglia homeostasis in the CNS [121]. In the context of CNS inflammation, microglia are important for astrocyte activation. In a sepsis model, microglia can polarize reactive astrocytes by releasing *IL-1α*, *TNF-α*, and *C1q,* which activates caspase-2/-3 signaling in neurons, leading to apoptosis and neurological damage [8]. In contrast, microglia can also signal astrocytes through molecules like *TGF-β*, which helps balance and protect against reperfusion injury in ischemic stroke [122].

Compared to non-tumor inflammatory diseases, the acquisition of astrocyte reactivity in the context of glioma possesses its unique mechanism. Glioma cell secretory products can directly induce *GFAP-*positive reactive astrocytes, and models of glioma-associated reactive astrocytes have been constructed in multiple studies based on this understanding [20,45,80]. Microglia primarily facilitate the responsiveness of astrocytes. Henrik et al. revealed that the formation of *GFAP+CD274+*-reactive astrocytes, which create an immunosuppressive microenvironment, relies on activating the JAK/STAT signaling pathway. The depletion of microglia in a glioma model in human brain slices resulted in a significant decrease in the activity of the astrocyte JAK/STAT signaling pathway, which was accompanied by a downregulation of *GFAP* and *CD274* expression [28]. Also, reactive astrocytes can influence the microglia phenotype. Perelroizen R et al. pointed out that reactive astrocytes can recruit microglia/TAMs and give them a pro-tumorigenic phenotype by upregulating the expression of *CCL2* and *CSF1* [27]. In addition, a multilateral cytokine interaction network exists among microglia, astrocytes, and tumor cells. Yao et al. found that medulloblastoma stimulated *IL-4* secretion from reactive astrocytes in a STAT6-dependent manner. Microglia receiving *IL-4* signals upregulate the activity of the JAK/STAT and MAPK/ERK signaling pathways and secrete *IGF1*, promoting tumor cell growth [44]. In summary, microglia could be important regulators of the acquisition of responsiveness by astrocytes and functionally synergize or complement with astrocytes in promoting the malignant progression of gliomas. These findings provide new insights and ideas for the research of the glioma microenvironment.

### 5.4. Reactive Astrocyte–Circulating Immune Cell Interactions

During the formation of gliomas, many circulating immune cells are recruited into the tumor tissue with the disruption of the blood–brain barrier. Cytotoxic cells, represented by CD8+ T cells and NK cells, exert a major tumor-lysogenic capacity. However, gliomas evolve to initiate mechanisms of immune escape, frequently manifest as cytotoxic cell depletion. This largely depends on the widespread presence of immunosuppressive effector molecules in the glioma microenvironment [123]. Among the available evidence, glioma-reactive astrocytes can secrete a variety of cytokines and chemokines, including *IGF1*, *CCL20*, *TGF-β*, *IL-10*, *IL-12*, *IL1A/B*, *IL-2*, *CXCL10*, and *G-CSF* [24,28,44,45]. These cytokines and chemokines proved to be engaged in recruiting and polarizing specific circulating immune cells in glioma. For example, *IL-10* recruits Treg cells and enhances their function, promoting depletion of T cells within the tumor [92,93]. Similarly, *IL-2* has been shown to activate Treg cells residing in the CNS and function to reverse neuroinflammation. *TGF-β* is an important immunosuppressive molecule in the glioma microenvironment. *TGF-β* can induce the polarization of TAMs towards an M2-like phenotype by activating the *PI3K/AKT* signaling pathway, and it can also facilitate glioma cells’ evasion from NK cells through the αv integrin-mediated *TGF-β* signaling pathway [13,14]. Thus, based on circumstantial evidence, information interaction channels between reactive astrocytes and circulating immune cells seem to exist. However, there is no direct evidence to support this hypothesis in the context of glioma. These mechanisms remain need to be elucidated and refined in the future.

### 5.5. Reactive Astrocyte–Extracellular Matrix Interactions

CNS injury, inflammation, or tumor lesions are often accompanied by a remodeling process of the extracellular matrix (ECM). Astrocytes serve an important secretory function in the CNS and are a major source of ECM structure molecules [124]. In the pathological state, the acquisition of reactivity by astrocytes is followed by a marked change in the secretory function of ECM. After CNS injury, reactive astrocytes may form the glial scar by changing ECM secretion patterns, potentially serving as a protective precaution to limit further injury and inflammation spread [125,126]. Similarly, peri-cancerous astrocytes can form astrogliosis proliferation bands and encase glioma tissue. This may be related to specific pathologic changes in gliomas. For example, glioma stem cells can upregulate the expression of *MAO-B* and *MCT1* in astrocytes via the metabolite acetate or inhibit the activity of the p53 signaling pathway in astrocytes to induce astrogliosis [21,127]. In addition, impaired blood–brain barrier function causes the deposition of plasma-derived fibrinogen and fibronectin. These exogenous ECM proteins can activate the *TGF-β* signaling pathway in astrocytes, which in turn alters the expression and secretion of ECMs such as chondroitin sulfate [90,91]. However, whether the astrogliosis bands can limit glioma invasion and metastasis is currently unsettled. Among the current findings, the expression and secretion patterns of reactive astrocytes seem to indicate that they can contribute to glioma progression through ECM reorganization. *VIM* is one of the important upregulated markers of reactive astrocytes [128]. Also, *VIM* is an important marker for mesenchymal subtypes of glioma tissues, which are closely related to EMT, invasion, and metastatic processes [129,130]. Lu et al. found that tumor cells could induce astrocytes to acquire reactivity through the Wnt/β-catenin signaling pathway, as evidenced by a decrease in E-calmodulin expression and an increase in the expression of *VIM* and *MMPs*. They hypothesized that the conversion of astrocytes to a mesenchymal subtype could assist tumor cells in catabolizing the extracellular matrix, which in turn promotes invasion and metastasis [131]. Le et al. found that reactive astrocytes provide precursor proteins to *MMP2*, which are cleaved via the activation of fibrinolytic enzymes by the tumor cells to catabolize the extracellular matrix in the form of active MMP2 [17]. Thus, the astrogliosis bands in gliomas seem not to be dense but rather full of “channels”. Reactive astrocytes that acquire a mesenchymal-like phenotype may be one of the facilitators of migration and invasion.

Overall, reactive astrocytes are important regulators of the microenvironment and can potentially promote the malignant progression of gliomas.

## 6. Reactive Astrocytes in Glioma Treatment

Currently, the standard treatment for gliomas is surgical resection supplemented by TMZ chemotherapy and radiotherapy [132]. However, the repeated tendency of gliomas to recur and their susceptibility to develop resistance to radiation/chemotherapy pose a great challenge for their clinical management. Although novel glioma treatment strategies such as anti-angiogenic therapies, immunotherapies, epigenetic therapies, oncolytic viral therapies, and gene therapies have shown promising performance in preclinical trials, their actual performance in clinical trials is poor, and they are accompanied by risk or uncertainty of efficacy [133]. Here, we address the potential mechanisms and feasible coping strategies for reactive astrocytes to assist gliomas in generating radiation/chemotherapy resistance. At the same time, we discuss the feasibility of astrocyte-targeted therapies in gliomas and future directions based on advances in astrocyte-targeted therapies in CNS inflammatory diseases.

### 6.1. Reactive Astrocytes and Glioma Radiotherapy/Chemotherapy Resistance

As we mentioned above, intensive material and information exchange exists between reactive astrocytes and glioma cells, leading to the development of resistance to radiotherapy and chemotherapy through various mechanisms. Astrocytes can connect with gap junction connections on the glioma surface through *CX43*, significantly reducing apoptosis in glioma cells induced by the temozolomide and vincristine [29]. The mRNA of *MGMT* can be delivered from reactive astrocytes to tumor cells via exosomes, which enhances their resistance to TMZ-induced apoptosis [80]. Also, upregulated expression of *IL1-β* by reactive astrocytes can stimulate the transformation of glioma-initiating cells to mesenchymal-like subtypes, leading to their therapeutic resistance [134]. In turn, radiation/chemotherapy can induce abnormally reactive astrocytes. Fletcher-Sananikone E et al. pointed out that stromal cells in the glioma microenvironment can acquire a senescence-associated secretory phenotype upon exposure to ionizing radiation, and this is particularly evident in astrocytes. Under such conditions, activated reactive astrocytes upregulate the expression of senescence factors such as *CDKN1A*, *HGF*, and *RTK*, increasing the invasiveness of glioma cells by activating the MET signaling pathway [32]. Berg et al. found that ionizing radiation can induce reactive astrocytes, which can promote glioma stem cell survival by upregulating *transglutaminase 2 (TGM2)* [30]. Targeting *TGM2* can significantly reduce tumor recurrence and post-radiotherapy cancer volume [30]. Also, the *TGM2-*related Gln-Glu cycle facilitates metabolite exchange between gliomas and astrocytes [46]. Moreover, the transfer of mitochondria from stromal cells to glioma cells might be related to the emergence of chemoresistance. This phenomenon has been reported in a study of microglia and GBM [135]. Similarly, mitochondrial transfer also occurs between reactive astrocytes and glioma cells [18]. Interestingly, Sun C et al. implanted astrocyte-derived mitochondria into gliomas, which instead increased their sensitivity to radiotherapy [135,136]. Thus, this topic has become controversial. We believe that a transient increase in the number of mitochondria within glioma cells may be beneficial for the radiotherapy effect. This is because mitochondria damaged by ionizing radiation can release apoptotic signals, which are conducive to the clearance of cancer cells. However, continuous mitochondrial delivery may be harmful, as it may endow the remaining glioma cells with stronger proliferative and invasive capabilities. In summary, these results have prompted us to wonder whether other mechanisms are involved in the exchange of substances and information between reactive astrocytes and gliomas that contribute to the development of therapeutic resistance. Can targeting these pathways enhance the effectiveness of radiation and chemotherapy, reduce glioma recurrence, and extend patient survival? Future research needs to address these questions.

### 6.2. Potential Strategies for Targeting Reactive Astrocytes in Gliomas

The key to effectively targeting reactive astrocytes is to precisely manipulate their phenotype in the microenvironment and peritumor, freeing them from their “pro-tumorigenic” state or enabling them to inhibit glioma growth. Currently, abundant information exists on reversing the astrocyte phenotype in CNS inflammatory and degenerative diseases, which can provide new insights into targeting therapeutic strategies for gliomas. Firstly, the NF-κB signaling pathway is crucial for regulating astrocyte reactivity [137]. Strategies targeting this pathway can significantly reduce the generation of *GFAP+* reactive astrocytes and alleviate inflammations in abnormal CNS events like stroke, cerebral hemorrhage, Alzheimer’s disease, and spinal cord injury [88,138,139,140]. Secondly, intervention in reactive astrocytes can lead to a reversal of their pro/anti-inflammatory phenotype. For example, *nicotinamide adenine dinucleotide-dependent deacetylase sirtuin-1 (SIRT1)* is a crucial regulator of the pro-inflammatory response in reactive astrocytes. Knockdown of SIRT1 in astrocytes can reduce T-cell infiltration in the CNS and elevate the ratio of macrophages/microglia with *IL-10* secretion, manifested in the conversion of reactive astrocytes from pro-inflammatory to anti-inflammatory phenotype [141]. Although these studies aimed to suppress the aberrant inflammatory response in CNS, they offer inspiration for the development of targeted therapies for glioma-reactive astrocytes. Under the selection of appropriate molecules or pathways, artificial intervention into reactive astrocytes is achievable. This might potentially fulfill the conversion of glioma-reactive astrocytes possessing tumor-promoting capabilities into those astrocytes capable of normally reacting to anti-tumor immunity. To fulfill this vision, we need to understand the generation and inside regulatory mechanisms of glioma-reactive astrocytes. Research on this topic will be valuable for the development of astrocyte-targeted therapies.

Second, regarding effects, extensive material information interaction pathways exist between astrocytes and glioma cells. Key enzymes of the lactate, acetate, and Gln/Glu metabolic cycles and gap junction proteins such as *CX43* and *GAP43* that act as bridges of material and information can serve as potential targets for intervention within astrocytes [18,19,20,21,46,66,69,83]. Among them, the exploration of blockade therapies targeting the metabolic loops between gliomas and microenvironment cells has received much attention. The glutamine–glutamate cycle is a suitable site. Intervening in its synthesis and transport processes may be effective for gliomas. For example, Zhong et al. mentioned that *alanine-serine-cysteine transporter 2 (ASCT2)* is a key glutamine transporter. Inhibiting the activity of *ASCT2* can significantly reduce the uptake of glutamine by glioma cells, directly inhibit the growth of GBM, and decrease its resistance to TMZ therapy [142]. TGM2 is a key enzyme in glutamate metabolism. Inhibiting the activity of *TGM2* can significantly enhance the sensitivity of GBM to radiotherapy [30,143]. The enzyme glutamine synthetase (GS), which is abundant in astrocytes, catalyzes the conversion of glutamate to glutamine. Also, glutamine is related to the onset of glioma-related epilepsy [144]. Targeting the glutamate–glutamine cycle holds the potential not only to effectively impede the growth of gliomas but also to substantially reduce the incidence of epilepsy. This is beneficial for enhancing the quality of life for patients. Similarly, targeted therapies against the *AQP4* molecule may also have the effect of inhibiting the growth of gliomas and improving clinical symptoms. As mentioned above, the dysregulation of the *AQP4* molecule may be related to cerebral edema, increased intracranial pressure, headache, and cognitive impairment. In gliomas, *AQP4* has additional pathophysiological functions beyond its role as water channels. These functions are associated with glioma invasion, angiogenesis, and malignant progression [145,146]. Therefore, *AQP4* is also a promising target for clinical intervention. In addition, targeted therapy against the angiogenic capacity of glioma-reactive astrocytes is also a good option. VM can facilitate gliomas in obtaining blood supply and initiating invasion [98,99]. Despite cell adhesion molecules are important in the process of VM, *VEGF* signaling is indispensable in the formation of blood flow channel-like structures by cancer cells [100,108]. Reactive astrocytes are an important source of *VEGF* [107]. Inhibiting their release of *VEGF* may be effective in the treatment of gliomas. Table 1 compiles and presents the potential glioma treatment strategies currently reported, which center around the intervention of astrocytes. However, these studies may still be in the stage of preclinical research or ongoing clinical trials, and their specific mechanisms, as well as their actual performance and safety, remain to be further explored and evaluated. It is also unclear whether altering astrocyte metabolism impacts normal neuronal functions or whether shifting anti-inflammatory phenotypes to pro-inflammatory ones could hasten the recruitment of pro-tumorigenic immune cells to the microenvironment. These issues need to be identified and overcome in the future.

Overall, reactive astrocytes have non-negligible biological significance in microenvironment formation, metabolic regulation, pro-carcinogenic signaling, and acquisition of radiotherapy/chemotherapy resistance in gliomas. Targeting reactive astrocytes is a novel and promising strategy for the clinical management of gliomas that warrants further exploration in the future.

## 7. Conclusions

Astrocytes, an important component of the glioma microenvironment, are increasingly recognized as significant drivers of glioma progression. Reactive astrocytes can directly interact with tumor cells, enhancing malignancy and therapeutic resistance, and can also promote immune evasion, invasion, and metastasis through microenvironment remodeling. However, the pathophysiological mechanisms associated with reactive astrocytes are relatively advanced in non-tumor diseases, but there are still many imperfections in the context of glioma. Also, there are still large blanks regarding the heterogeneity of reactive astrocytes in the glioma microenvironment. Further understanding of the interacting mechanisms between astrocytes and gliomas will open new avenues for translational medicine and more meaningful clinical treatments for gliomas.

## Figures and Tables

**Table 1:** Currently reported glioma treatment strategies involving astrocyte targeting.

Intervention Method	Intervention Target	Mechanisms/Models	Reporters	Citations
Knockdown of ABCA1	ABCA1	Cholesterol serves as a pivotal metabolite underpinning the survival and proliferation of glioma cells. The approach of targeting ABCA1 curbs the growth of GBM by impeding the efflux of cholesterol from astrocytes. ***Mus musculus.***	Perelroizen R et al.	[27]
Galunisertib	TGF-β1	Astrocytes can promote the formation of VM in GBM via TGF-β1. Galunisertib can reduce VE-cadherin and smooth muscle actin-α expression by targeting and inhibiting the TGF-β1 molecule. By downregulating VM, Galunisertib inhibits the proliferation and invasion of GBM. ***Mus musculus.***	Zhang C et al.	[147]
Targeting GSC exosomes	miR-3065-5p/DLG2	miR-3065-5p is present in the exosomes of GSCs. It can promote astrocytes to acquire reactivity through the miR-3065-5p/DLG2 axis, thereby supporting the growth of glioma cells. Targeted inhibition of the miR-3065-5p/DLG2 axis can significantly downregulate the reactivity of astrocytes in the co-culture system with GSCs, achieving the function of indirectly inhibiting the growth of glioma. ***Homo sapien cell lines.***	Li H et al.	[148]
Knockdown/directed mutation of CX43	CX43	In astrocytes, shRNA-mediated intervention, overexpression of the dominant-negative channel-defective CX43-T154A mutant and replacement of the wild-type with a C-terminal truncated CX43 mutant can all prevent the formation of CX43 gap junctions between astrocytes and GBM, which can significantly inhibit the invasion of glioma cells. ***Mus musculus.***	Sin WC et al.	[19]
HGF-neutralizing antibodies/Crizotinib/ ABT-263 (navitoclax)	HGF/Tyrosine Kinase/senescent astrocytes	Astrocytes will enter the senescence state after being exposed to ionizing radiation, which is characterized by the upregulation of CDKN1A (p21) and the increased secretion of HGF and RTK. This can promote the migration and invasion of gliomas. Antagonizing HGF and inhibiting the tyrosine kinase activity can significantly reduce the tumor-promoting effects of astrocytes. Moreover, ABT-263 is an inhibitor of cellular senescence and can kill astrocytes that have entered the senescence state. These senolytic therapies can inhibit the growth and invasiveness of recurrent GBM. ***Mus musculus.***	Fletcher-Sananikone E et al.	[32]
β-hydroxybutyrate	Astrocytes	This study indicates that a ketogenic diet can inhibit the growth of glioma. The related metabolite β-hydroxybutyrate can promote the transformation of astrocytes into a pro-inflammatory phenotype, thereby inhibiting the growth of glioma. ***Mus musculus.***	de Ruiter Swain J et al.	[149]
AS1411	P65	AS1411 inhibits the entry of the transcription factor P65 of the NF-κB pathway into the nucleus and then downregulates the expression of miRNA-27a in astrocytes and exosomes. This can upregulate the expression of the miRNA-27a target gene INPP4B in gliomas, thereby inhibiting the PI3K/AKT pathway and suppressing glioma proliferation. ***Homo sapiens(clinical samples)* and *Mus musculus.***	Sun X et al.	[150]

## Data Availability

Not applicable.

## Abbreviations

The following abbreviations are used in this manuscript:

GBM	glioblastoma
LGG	lower-grade glioma
GFAP	glial fibrillary acidic protein
TGF-β	transforming growth factor-β
SPARC	acid secretory protein
snRNA-seq	single nuclear RNA sequencing
scRNA-seq	single-cell RNA sequencing
CNS	central nervous system
GJs	gap junctions
IFN-α	interferon-α
TNF	tumor necrosis factor
MGMT	O6-alkylguanine DNA alkyl transferase
TMZ	temozolomide
Gln	L-glutamine
Glu	glutamate
TCA	tricarboxylic acid
EMT	epithelial–mesenchymal transition
GSCs	glioma stem cells
VM	vasculogenic mimicry
ITGB8	β8 integrin
GS	glutamine synthetase
AQP4	aquaporin 4
ROS	reactive oxygen species
AD	Alzheimer’s disease
PD	Parkinson’s disease
ECM	extracellular matrix
TGM2	transglutaminase 2
OPC-like	oligodendrocyte progenitor cell-like
NPC-like	neuronal progenitor cell-like
AC-like	astrocyte-like
MES-like	mesenchymal-like
VIM	vimentin
ASCL1	achaete-scute complex-like 1
EGFR	epidermal growth factor receptor
OLIG2	oligodendrocyte transcription factor 2
MKI67	proliferating cell nuclear antigen Ki-67
PTEN	phosphatase and tensin homolog
TP53	tumor protein p53
RB1	retinoblastoma 1
SOX2	sex-determining region Y-box 2
S100B	s100 calcium-binding protein B
S100A10	s100 calcium-binding protein A10
C1q	complement component 1 q subcomponent
NES	neuroepithelial stem cell protein
VIM	vimentin
C3	complement component 3
MMP-2	matrix metallopeptidase 2
PI3K	phosphatidylinositol 3 -kinase
Akt	protein kinase B
Src kinase	Rous sarcoma virus-related tyrosine-protein kinase src
cGAMP	cyclic guanosine monophosphate-adenosine monophosphate
STRT1	nicotinamide adenine dinucleotide-dependent deacetylase sirtuin-1
ASCT2	alanine-serine-cysteine transporter 2
